# Dark/Light Treatments Followed by γ-Irradiation Increase the Frequency of Leaf-Color Mutants in *Cymbidium*

**DOI:** 10.3390/plants9040532

**Published:** 2020-04-20

**Authors:** Sang Hoon Kim, Se Won Kim, Jaihyunk Ryu, Si-Yong Kang, Byoung-Cheorl Kang, Jin-Baek Kim

**Affiliations:** 1 Advanced Radiation Technology Institute, Korea Atomic Energy Research Institute, Jeongeup 56212, Korea; shkim80@kaeri.re.kr (S.H.K.); sewonk@korea.kr (S.W.K.); jhryu@kaeri.re.kr (J.R.); sykang@kaeri.re.kr (S.-Y.K.); 2 National Institute of Agricultural Sciences, Rural Development Administration, Jeonju 54874, Korea; 3 Department of Plant Science, Plant Genomics and Breeding Institute, and Vegetable Breeding Research Center, College of Agriculture and Life Sciences, Seoul National University, Seoul 08826, Korea; bk54@snu.ac.kr

**Keywords:** chlorophyll, dark/light treatments, γ-ray, mutation, *Cymbidium*, leaf-color

## Abstract

Radiation randomly induces chromosomal mutations in plants. However, it was recently found that the frequency of flower-color mutants could be specifically increased by upregulating anthocyanin pathway gene expression before radiation treatments. The mechanisms of chlorophyll biosynthesis and degradation are active areas of plant study because chlorophyll metabolism is closely connected to photosynthesis. In this study, we determined the dark/light treatment conditions that resulted in upregulation of the expression levels of six chlorophyll pathway genes, uroporphyrinogen III synthase (*HEMD*), uroporphyrinogen III decarboxylase (*HEME2*), NADPH-protochlorophyllide oxidoreductase (POR) A (*PORA*), chlorophyll synthase (*CHLG*), chlorophyllase (*CLH2*), and red chlorophyll catabolite reductase (*RCCR*), and measured their effects on the γ-irradiation-induced frequencies of leaf-color mutants in two *Cymbidium* cultivars. To degrade chlorophyll in rhizomes, 60–75 days of dark treatment were required. To upregulate the expressions of chlorophyll pathway genes, 10 days of light treatment appeared to be optimal. Dark/light treatments followed by γ-irradiation increased chlorophyll-related leaf mutants by 1.4- to 2.0-fold compared with γ-ray treatment alone. Dark/light treatments combined with γ-irradiation increased the frequency of leaf-color mutants in *Cymbidium*, which supports the wider implementation of a plant breeding methodology that increases the mutation frequency of a target trait by controlling the expression of target trait-related genes.

## 1. Introduction

Physical mutagens, such as X-rays [1,2,3], γ-rays [4,5,6], and ion particles [1,3,7,8,9], and chemical mutagens, such as ethyl methanesulfonate [1,3,10,11,12] and *N*-nitroso-*N*-methylurea [13,14,15], have been widely used to induce mutations in various plants. In plant mutation breeding, researchers have focused on developing methods to increase the mutation frequency and broaden the mutation spectrum. Three strategies have been explored to achieve these objectives: first, controlling the irradiation conditions, such as total dose [4,16,17,18], dose rate [19,20,21], irradiation duration [22,23], and aerospace environment [24,25]; second, controlling the material conditions, such as developmental stage [26] and plant tissues [27,28]; and third, using different radiation types, such as heavy ion particles [16,29,30] and proton ion particles [9,31]. Generally, radiation induces mutations randomly in plant chromosomes.

Recently, it was reported that in human cells the sensitivity of DNA to γ-irradiation varied with the chromatin status [32,33]. Takata et al. [32] reported that the ratio of DNA double-strand breaks to γ-irradiation was higher in decondensed chromatin than in condensed chromatin, and Venkatesh et al. [33] verified that sensitivity to γ-irradiation, in terms of DNA double-strand breaks, was higher in euchromatin than heterochromatin regions. Additionally, Hase et al. [34] suggested that radiation could increase the mutation frequency for flower color when the genes involved in flower-color synthesis are highly expressed. The effect of sucrose treatment on the expression of anthocyanin pathway genes, an important mechanism for altering flower color [35], has been demonstrated in Arabidopsis [36]. A sucrose treatment followed by a radiation treatment increases the frequencies of flower-color mutants in chrysanthemum and petunia, although no anthocyanin pathway gene expression levels were measured [28,34]. Recently, Kim et al. [37] demonstrated that sucrose and methyl jasmonate treatments increase the frequency of flower-color mutants induced by γ-irradiation in chrysanthemum, which confirms the upregulated expressions of several anthocyanin pathway genes.

For floricultural crops, flower and leaf color are among the most important characteristics that determine commercial value in the flower market. In particular, variegation of leaves in foliage plants is more important than that of flowers. In rice, the variegation of leaves has been extensively studied to understand chlorophyll (Chl) biosynthesis and degradation, chloroplast development, and photosynthesis [38]. To date, more than 50 leaf-color-related genes have been cloned in rice (*Oryza sativa*) [38,39]. It was reported that mutations of 13 genes (*gra75*, *OsCAO*, *OsCHLD*, *OsCHLH*, *OsChlI*, *OsDET1*, *OsDVR*, *OsGluRS*, *OsPORB*, *lyl1-1*, *sdl*, *ygl1*, and *ygl7*) in the Chl biosynthesis pathway and five genes (*sgr*, *nol*, *nyc1*, *nyc3*, and *nyc4*) in the Chl degradation pathway caused phenotypic variations in rice [38,39,40,41]. Chls are synthesized instantly upon exposure to light [42] and, in association with photosystem II, absorb light energy to drive essential photochemistry in photosynthesis [43]. In plants, there are two forms of Chls, Chl *a* and *b* [42]. Zhu et al. [42] reported that phytohormones, such as ethylene, abscisic acid, and jasmonic acid, and light affect Chl degradation. Additionally, it was reported that salicylic acid [44] and brassinolide [45] are promoters, while cytokinin [46] and gibberellic acid [47] are repressors, of Chl degradation.

In this study, we determined the dark/light treatment conditions that resulted in the induced upregulated expression levels of six genes associated with Chl metabolism and assessed their effects on the frequency of γ-irradiation-induced Chl-related leaf-color mutants in *Cymbidium*.

## 2. Results

### 2.1. Effect of Dark/Light Treatments on Chl Degradation and Biosynthesis

**Figure 1:**
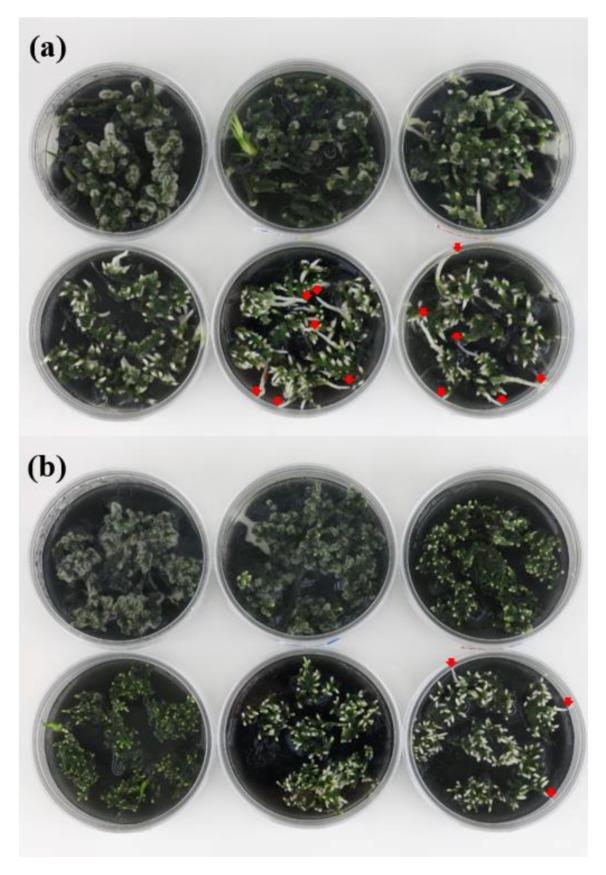
Chlorophyll degradation after dark treatment in rhizomes of *Cymbidium* hybrids RB003 and RB012. (**a**) RB003; (**b**) RB012. From the upper left: 0, 20, 40, 60, 75, and 90 days after dark treatment. Red arrows indicate the initial regeneration of the rhizomes.

To induce Chl biosynthesis, dark/light treatments were conducted on the rhizomes of two *Cymbidium* cultivars, RB003 and RB012. The dark treatment was first conducted to degrade Chls and was then followed by light treatment to induce Chl biosynthesis. Chl degradation was visually apparent on the terminal parts of rhizomes after 40 days of dark treatment (Figure 1). There were differences in Chl degradation caused by the dark treatment between the two cultivars because the rate of Chl degradation in RB003 rhizomes was higher than that in RB012 rhizomes. Consequently, the minimum durations of dark treatments observed to degrade Chls were 60 and 75 days for RB003 and RB012, respectively (Figure 1). For dark treatments that were longer than minimum durations, the regeneration of the rhizome terminal parts was more apparent than additional effects on Chl degradation (Figure 1).

**Figure 2:**
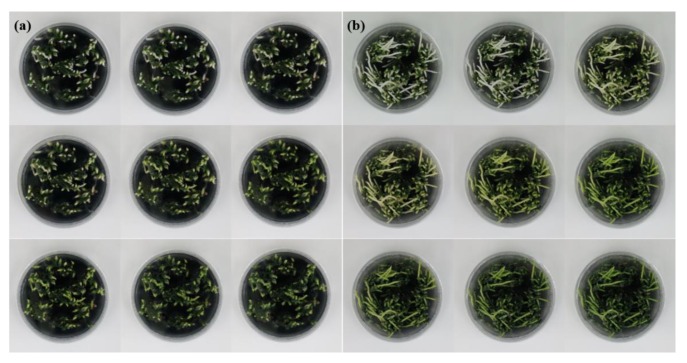
Chlorophyll accumulation after light treatment in rhizomes of *Cymbidium* hybrids RB003 and RB012. (**a**) RB003; (**b**) RB012. From the upper left: dark-treated rhizomes treated with light for 0, 1, 5, 7, 10, 14, 15, 20, and 21 days. Dark treatment durations were 60 and 75 days for the rhizomes of *Cymbidium* hybrids RB003 and RB012, respectively.

**Figure 3:**
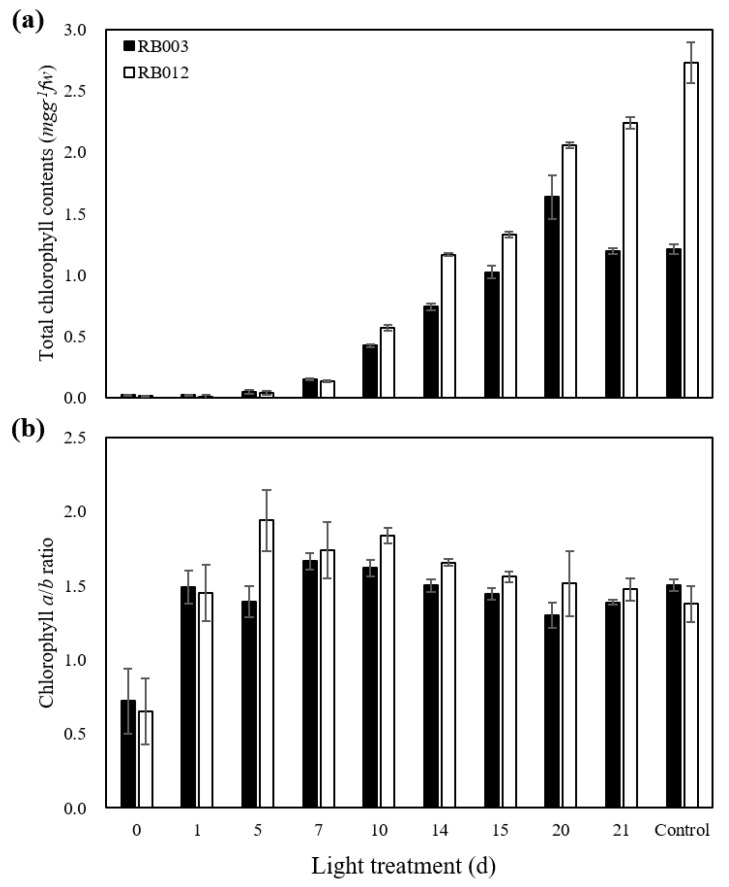
Chlorophyll contents after light treatment in rhizomes of *Cymbidium* hybrids RB003 and RB012. (**a**) Total chlorophyll contents; (**b**) Chlorophyll *a*/*b* ratio. Dark-treated rhizomes were subjected to light treatment. Dark treatment durations were 60 and 75 days for the rhizomes of *Cymbidium* hybrids RB003 and RB012, respectively.

Following dark treatment, Chl accumulations in rhizomes were observed after seven days of light treatment in both RB003 and RB012 (Figure 2). The Chl accumulation in response to light treatment was more clearly observable in RB012 rhizomes than in RB003 rhizomes. Chl levels increased rapidly after 10 days of light treatment in both cultivars (Figure 3a). The Chl level in RB003 rhizomes reached a maximum at 20 days of light treatment, decreased at 21 days, and reached a constant level that was similar to that of controls. For RB012 rhizomes, the Chl amount continuously increased until the 21st day of light treatment, but the level was less than that of the control (Figure 3a). The Chl *a*/*b* ratios in RB003 and RB012 increased until seven and five days of light treatment, respectively, and then decreased to the control levels (Figure 3b). Additionally, the Chl content of an RB012 control that was not subjected to the dark treatment was more than 2-fold higher than that of the RB003 control. The rates of Chl degradation and biosynthesis were higher in RB003 rhizomes than in RB012 rhizomes (Figure 1, Figure 2 and Figure 3).

### 2.2. Effect of Dark/Light Treatments on Chl Pathway Gene Expression

**Figure 4:**
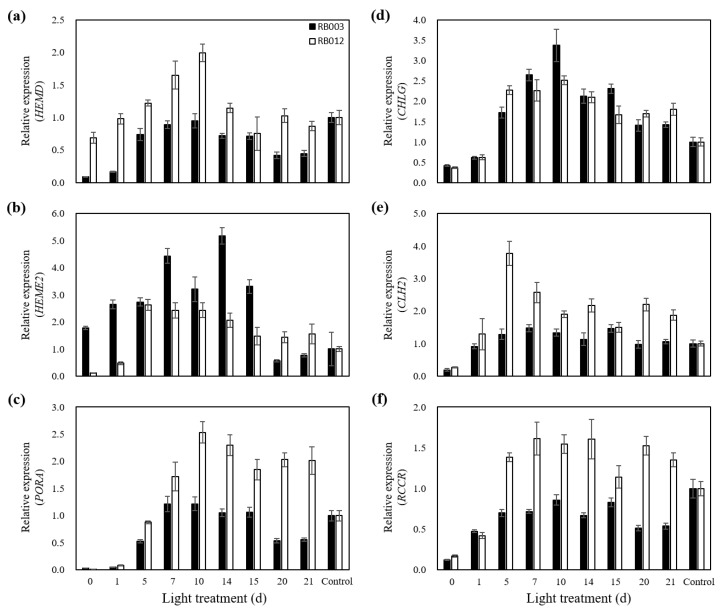
Relative expression of six genes involved in chlorophyll biosynthesis and degradation during light treatment of *Cymbidium* hybrids RB003 and RB012. (**a**) Relative expression of *HEMD*; (**b**) Relative expression of *HEME2*; (**c**) Relative expression of *PORA*; (**d**) Relative expression of *CHLG*; (**e**) Relative expression of *CLH2*; (**f**) Relative expression of *RCCR*. Dark-treated rhizomes were subjected to light treatment. Dark treatment durations were 60 and 75 days for the rhizomes of *Cymbidium* hybrids RB003 and RB012, respectively.

To identify the effects of dark/light treatments on Chl pathway gene expression levels, the expression patterns of four genes (*HEMD*, *HEME2*, *PORA*, and *CHLG*) involved in Chl biosynthesis and two genes (*CLH2* and *RCCR*) involved in Chl degradation were analyzed after various durations of light treatment that followed dark treatment (Figure 4). In the RB003 cultivar, the expression levels of *HEME2* and *CHLG* gradually increased to their highest points at 14 and 10 days after light treatment, respectively, and then decreased (Figure 4b,d). The expression levels of *HEMD*, *PORA*, and *RCCR*, but not *CLH2*, gradually increased until 10 days after light treatment and then decreased, although their expressions were not higher than in untreated controls (Figure 4a,c,f). In the RB012 cultivar, the expression levels of *HEMD*, *PORA*, and *CHLG* gradually increased until reaching their highest values after 10 days of light treatment and then decreased, as seen with RB003 (Figure 4a,c,d). The expression levels of *HEME2*, *CLH2*, and *RCCR* were highest at relatively early time points, after 5–7 days of light treatment (Figure 4b,e,f). However, the expression levels of *PORA*, *CHLG*, *CLH2*, and *RCCR* remained high, even after relatively long light treatments, which differed from their expression patterns in RB003 (Figure 4c–f). Overall, although the expression patterns of the six genes differed depending on the target gene and the cultivar, 10 days of light treatment appeared to be optimal for inducing the upregulated expression of Chl pathway genes.

### 2.3. Induction Frequency of Chl-Related Leaf-Color Mutants

**Figure 5:**
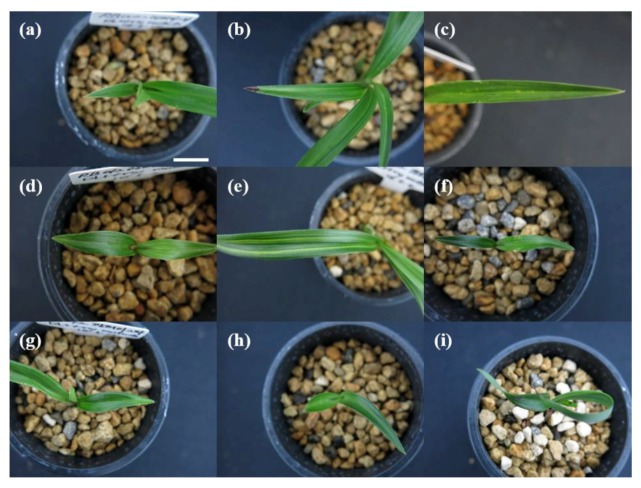
Representative leaf-color and -shape mutants induced by dark/light treatments followed by γ-irradiation in *Cymbidium* hybrids RB003 and RB012. (**a**–**c**) Mutants induced in *Cymbidium* hybrid RB003; (**d**–**i**) Mutants induced in *Cymbidium* hybrid RB012. (**a**,**c**) Yellow marginal leaf-color mutants; (**b**,**d**,**e**,**g**,**h**) Yellow stripe leaf-color mutants; (**f**) Dwarf leaf-shape mutant; (**i**) Abnormal leaf-shape mutant. Scale bar: 1 cm.

**Figure 6:**
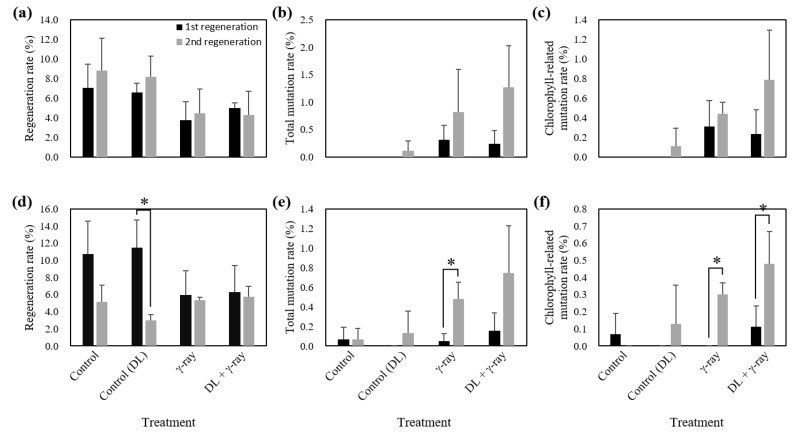
Regeneration, total mutation, and chlorophyll-related mutation rates of mutant populations induced by dark/light treatments followed by γ-irradiation in *Cymbidium* hybrids RB003 and RB012. (**a**–**c**) Regeneration, total mutation, and chlorophyll-related mutation rates in the RB003 population; (**d**–**f**) Regeneration, total mutation, and chlorophyll-related mutation rates in the RB012 population. DL, dark/light treatments. Student’s *t*-test was used to calculate statistical significance (* *p* < 0.05).

Dark/light treatments (dark treatment for 60 days (RB003) and 75 days (RB012) followed by light treatment for 10 days) followed by γ-irradiation (50 Gy (RB003) and 30 Gy (RB012)) were performed, and the phenotypes of RB003 and RB012 populations were analyzed to determine regeneration, total mutation, and Chl-related mutation rates (Figure 5 and Figure 6). Regeneration rates of γ-irradiated populations were reduced compared with those of the two control populations (RB003: Control, 7.9, Control (DL), 7.4, γ-ray, 4.1, DL + γ-ray, 4.6; RB012: Control, 8.0, Control (DL), 7.2, γ-ray, 5.7, DL + γ-ray, 6.0) (Figure 6a,d). Total mutation rates of dark/light treatments followed by γ-irradiated populations were greater than those of only γ-irradiated populations (RB003: γ-ray, 0.56, DL + γ-ray, 0.75; RB012: γ-ray, 0.26, DL + γ-ray, 0.45) (Figure 6b,e). Additionally, somaclonal variations without γ-irradiation were also identified at a low frequency (RB003: Control (DL), 0.05; RB012: Control, 0.07, Control (DL), 0.07) (Figure 6b,e). Chl-related mutation rates of dark/light treatments followed by γ-irradiated populations were greater than those of only γ-irradiated populations (RB003: γ-ray, 0.37, DL + γ-ray, 0.51; RB012: γ-ray, 0.15, DL + γ-ray, 0.30), although the difference was not statistically significant (Figure 6c,f). Interestingly, in both cultivars, relatively more mutants were identified in the second regenerated populations than in the first regenerated populations (Figure 6).

## 3. Discussion

### 3.1. Dark/Light Treatments Upregulate Gene Expression in the Chl Pathway

Chl plays important light-harvesting and energy transduction roles in photosynthesis, and almost every gene involved in Chl biosynthesis and degradation has been identified [48]. Chl biosynthesis and degradation can be summarized as follows [48]: biosynthesis, glutamyl tRNA followed by 5-aminolevulinic acid (ALA), protoporphyrin IX (Proto), protochlorophyllide *a* (Pchlide *a*), chlorophyllide *a* (Chlide *a*), and Chl *a*; degradation, Chl *a* followed by Chlide *a*, Pchlide *a*, red Chl catabolite (RCC), primary fluorescent Chl catabolite (pFCC), and nonfluorescent Chl catabolites.

In this study, we analyzed time-course expression levels of four biosynthesis pathway genes (*HEMD*, *HEME2*, *PORA*, and *CHLG*) and two degradation pathway genes (*CLH2* and *RCCR*) during light treatments that followed the dark treatment (Figure 4). *HEMD* and *HEME2* function in the biosynthesis of ALA to Proto as follows [48]: *HEMD*, uroporphyrinogen III synthase, cyclizes and isomerizes hydroxymethylbilane to produce uroporphyrinogen III in the plastid [49] and *HEME2*, uroporphyrinogen III decarboxylase, eliminates the carboxyl groups from the four acetate side chains of uroporphyrinogen III to produce coproporphyrinogen III [50]. *PORA* and *CHLG* function in the biosynthesis of Pchlide *a* to Chl *a* as follows [48]: *PORA*, NADPH-protochlorophyllide oxidoreductase (POR) A, catalyzes a light-dependent trans-reduction of the D-ring of Pchlide *a* to produce Chlide *a* in the plastid membranes [51] and *CHLG*, Chl synthase, catalyzes the esterification of Chlide *a* to produce Chl *a* in the plastid membranes [52]. *CLH2* and *RCCR* function in the degradation of Chl *a* to Chlide *a* and RCC to pFCC, respectively [48], as follows: *CLH2*, chlorophyllase, catalyzes the hydrolysis of Chl *a* to produce Chlide *a* in the plastid or vacuole [53] and *RCCR*, red Chl catabolite reductase, catalyzes the cleavage of RCC to produce pFCC in the plastid or stroma [54]. Eckhardt et al. [48] reported that the expression of most genes involved in Chl biosynthesis are light-inducible and developmental stage-dependent, while Chl degradation genes, except Pchlide *a* oxygenase, are constitutively expressed. In RB012, all four genes involved in Chl biosynthesis were highly expressed after light treatment (Figure 4a–d), which is consistent with a previous report; however, both genes involved in Chl degradation were also highly expressed after light treatment (Figure 4e,f). In RB003, only two Chl biosynthesis genes, *HEME2* and *CHLG*, were highly expressed after light treatment (Figure 4b,d). Overall, there were differences between expression responses of Chl pathway genes in this study and those of a previous report [48], which may result from the different plant species being studied. It has been reported that when the Chl accumulation in dark-grown seedlings under light treatment conditions has reached a maximum level, the POR activity decreases to an undetectable level [55]. In this study, the relative expression levels of the six genes studied reached maximums before the maximum Chl accumulation was reached (Figure 3 and Figure 4).

### 3.2. Dark/Light Treatments Followed by γ-Irradiation Increase the Frequency of Leaf-Color Mutants

In previous reports, effects of sucrose and plant hormones on the expression of anthocyanin pathway genes were definitively demonstrated in Arabidopsis [36,56]. Using a sucrose pre-treatment to upregulate anthocyanin biosynthesis gene expression, Hase et al. [34] and Kim et al. [22] reported that the sucrose pre-treatment followed by carbon-ion and γ-ray treatments resulted in 2.5-fold and 2- to 10.5-fold increases, respectively, in the frequencies of flower-color mutants in petunia [34] and chrysanthemum [22]. Additionally, Kim et al. [37] elucidated the optimal pre-treatment conditions (50 mM sucrose with 100 μM methyl jasmonate for 18 h) and measured the effects of pre-treatment on the expression patterns of six anthocyanin pathway genes. They reported that the sucrose with methyl jasmonate pre-treatment followed by γ-irradiation resulted in a 1.5-fold increase in the frequency of flower-color mutants in chrysanthemum [37].

Following the successful demonstration that the frequency of flower-color mutants could be increased by the upregulation of anthocyanin pathway gene expression, we applied a similar methodology to increase the frequency of leaf-color mutants in *Cymbidium*. In the present study, dark/light treatments followed by γ-irradiation resulted in 1.4-fold (RB003) and 2.0-fold (RB012) increases in the rates of Chl-related leaf-color mutations compared with those of γ-ray treatment alone, although a statistically significant difference was not confirmed (Figure 6c,f). Presumably, this results from differences in mechanism, complexity, and the number of genes that determine the colors in flowers and leaves in anthocyanin and Chl pathways [35,36,57]. Additionally, there are 19 types of anthocyanins [35], but only two types of Chls (Chl *a* and *b*) in higher plants [42]. Furthermore, in the second regenerated populations, relatively more mutants were identified, which may result from the time needed for the stabilization, expansion, and competition of mutated cells compared with normal cells [58]. Therefore, additional experiments will be necessary to establish optimal conditions for upregulating Chl pathway gene expression to increase mutation rates. Regardless, the present study supports the use of a methodology that increases the mutation frequency of a target trait by controlling the expression of target trait-related genes.

## 4. Materials and Methods

### 4.1. Plant Materials

In this study, two *Cymbidium* hybrid (*Cymbidium sinense* × *Cymbidium goeringii*) cultivars, RB003 and RB012, were used. Rhizomes of those two cultivars were cultured at 24 ± 1 °C with a 16-h photoperiod provided by white fluorescent lights (PPFD = 50 μmol m^−2^ s^−1^) on a medium (pH 5.35) comprising 0.1% Hyponex (N:P:K = 20:20:20; Hyponex Japan Co., Ltd., Osaka, Japan), 0.2% Hyponex (N:P:K = 6.5:6:19), 0.3% peptone (Duchefa B.V., Haarlem, The Netherlands), 3% sucrose (Duchefa B.V.), 0.38% plant agar (Duchefa B.V.), and 0.075% activated charcoal (Sigma-Aldrich, St Louis, MO, USA).

### 4.2. Dark/Light Treatments

One-month-old rhizomes were used for dark/light treatments of both cultivars. Dark/light treatments were conducted as follows: to degrade the Chl in the rhizomes, the dark treatment was conducted for 0, 20, 40, 60, 75, or 90 days; next, to synthesize Chl in the rhizomes, the light treatment with a 16-h photoperiod (the same as the culture condition) was conducted for 0, 1, 5, 7, 10, 14, 15, 20, or 21 days.

### 4.3. Chl Analysis

Rhizomes treated with dark/light conditions were sampled for Chl content analysis. The amounts of Chl *a* and *b* were estimated using the method of Lichtenthaler [59]. Rhizomes were ground in liquid nitrogen, and the pigments were extracted in 95% ethanol (Sigma, St. Louis, MO, USA). The extract was vortexed for 24 h at room temperature in the dark. After centrifugation, the absorbance of the supernatants was quantitatively measured with a UV-1800 spectrometer (Shimadzu, Kyoto, Japan) at 664.2 nm, 648.6 nm, and 470 nm. Experiments were performed as three replicates.

### 4.4. RNA Extraction and RT-qPCR Analysis

Total RNA was isolated from the dark/light-treated rhizomes using an RNeasy Plant Mini Kit (Qiagen, Hilden, Germany). The concentration and quality of the extracted RNA were assessed using a Nanodrop 2000 spectrophotometer (Thermo Fisher Scientific, Waltham, MA, USA). First-strand cDNA synthesis was conducted using a ReverTra Ace-α kit (Toyobo Co. Ltd., Osaka, Japan). Reverse transcription quantitative PCR (RT-qPCR) was conducted with iQ SYBR Green Supermix (Bio-Rad, Hercules, CA, USA) using the CFX96 Touch Real-Time PCR Detection System (Bio-Rad, Hercules, CA, USA). RT-qPCR was performed following the method of Kim et al. [60]. Transcript levels of each gene were normalized to those of *Actin*. Three experimental replicates were performed. The primer sequences used for RT-qPCR are listed in Table S1.

### 4.5. γ-ray Treatments and Evaluation of Induced Leaf Mutants

Dark/light-treated rhizomes (dark treatment for 60 days (RB003) and 75 days (RB012) followed by light treatment for 10 days) of two cultivars were γ-irradiated (50 Gy for RB003, and 30 Gy for RB012) using a ^60^Co source (150 TBq capacity; AECL, Canada) for 24 h at the Korea Atomic Energy Research Institute, Jeongeup, Korea. Experiments were performed with three biological replicates and 250 rhizomes per treatment per replicate.

Phenotype analysis of γ-irradiated RB003 and RB012 populations was conducted twice, at 6 and 10 months after γ-ray treatment, to measure regeneration, total mutation, and Chl-related mutation rates.

### 4.6. Statistical Analyses

Student’s *t*-tests were used to compare the significance of differences in means among treatments. The *p*-value was considered to be statistically significant at the 0.05 significance level.

## Supplementary Materials

The following are available online at https://www.mdpi.com/2223-7747/9/4/532/s1, Table S1: Primers for RT-qPCR analysis.
