# Supplementary material for: Dark/Light Treatments Followed by γ-Irradiation Increase the Frequency of Leaf-Color Mutants in Cymbidium
Source: Plants (Basel). 2020 Apr 20;9(4):532. doi: 10.3390/plants9040532 (PMC7238429; doi:10.3390/plants9040532)
Supplement: Supplementary file 1 [file plants-09-00532-s001.pdf]

Supplementary Materials

Table S1. Primers for RT-qPCR analysis.

ID	Sequence (5′ to 3′)
HEMD-F	CTTCGCCTCTGCTTCTCCT
HEMD-R	TGCCAGCACCAACAACTCC
HEME2-F	TTATCGAGAACGCCCGTTT
HEME2-R	TGTACCTCCCTGCTTGCCT
PORA-F	GCCTCCTCTTTCCTCGCAC
PORA-R	GGCTGTTGCTGTCGTCTGG
CHLG-F	GTCTCAGTCGCCGTCTCGA
CHLG-R	TCTTCCATTTGTCCGTGCC
CLH2-F	CATGGCTCCACCAGCAAAA
CLH2-R	CCTCCTTGTGCTCCCAAGG
RCCR-F	TTCACACCGCCTCTCATCA
RCCR-R	AACCGAGGCGATCGTCAAC
Actin-F	AATCCCAAGGCAAACAGA
Actin-R	CCATACCAGAATCCAG
